# Synthesis and Biodegradation of Poly(l-lactide-*co*-β-propiolactone)

**DOI:** 10.3390/ijms18061312

**Published:** 2017-06-20

**Authors:** Yuushou Nakayama, Kazuki Aihara, Zhengguo Cai, Takeshi Shiono, Chikara Tsutsumi

**Affiliations:** 1 Department of Applied Chemistry, Graduate School of Engineering, Hiroshima University, Higashi-Hiroshima 739-8527, Japan; kazuki.a.1988@gmail.com; 2 State Key Laboratory for Modification of Chemical Fibers and Polymer Materials, College of Material Science and Engineering, Donghua University, Shanghai 201620, China; caizg@dhu.edu.cn; 3 Department of Applied Chemistry and Biotechnology, Niihama National College of Technology, Niihama 792-8580, Japan; tsutsumi@chem.niihama-nct.ac.jp

**Keywords:** copolymerization, l-lactide, β-propiolactone, trifluoromethanesulfonic acid, poly(l-lactide-*co*-β-propiolactone), thermal properties, biodegradation

## Abstract

Although the copolymerizations of l-lactide (LA) with seven- or six-membered ring lactones have been extensively studied, the copolymerizations of LA with four-membered ring lactones have scarcely been reported. In this work, we studied the copolymerization of LA with β-propiolactone (PL) and the properties of the obtained copolymers. The copolymerization of LA with PL was carried out using trifluoromethanesulfonic acid as a catalyst and methanol as an initiator to produce poly(LA-*co*-PL) with *M*_n_ of ~50,000 and PL-content of 6–67 mol %. The *T*_g_ values of the copolymers were rapidly lowered with increasing PL-contents. The *T*_m_ and Δ*H*_m_ of the copolymers gradually decreased with increasing PL-contents, indicating their decreased crystallinity. Biodegradation test of the copolymers in compost demonstrated their improved biodegradability in comparison with the homopolymer of LA.

## 1. Introduction

Aliphatic polyesters such as poly(l-lactic acid) (or poly(l-lactide), PLA), poly(ε-caprolactone) (PCL), and poly(β-hydroxyalkanoate)s (PHAs) such as poly(β-hydroxybutyrate) (PHB) are known as typical biodegradable polymers [1,2,3,4,5,6,7]. PLA is one of the most promising biodegradable polymers with a glass transition temperature (*T*_g_) of ca. 60 °C and a melting temperature (*T*_m_) of ca. 170 °C. PLA is known for its renewability, biocompatibility, and high rigidity, and thus has been utilized for biomedical, pharmaceutical, and agricultural applications as well as commodity applications [4,5]. However, biotic degradations or degradations in the natural environment of polylactides tend to be relatively slow among the biodegradable polyesters [8,9,10]. In order to improve the degradability of PLA, several copolymers of l-lactide (LA) with other cyclic esters such as ε-caprolactone (CL) and blends of PLA with other polyesters have been extensively studied [8,9,11,12,13].

PHAs are known for their good biodegradability [14,15]. PHAs including PHB were originally produced by fermentation with some bacteria [14,15], while the ring-opening polymerization of the corresponding four-membered ring lactones such as β-butyrolactone (BL) also gives the structurally same polymers with PHAs [16,17]. Unsubstituted four-membered ring lactone, β-propiolactone (PL), can also be subjected to ring-opening polymerization to form poly(β-propiolactone) (PPL) [16], which is known to show excellent degradability [18,19,20,21]. PPL, a structural isomer of polylactide, typically shows *T*_g_ at around −20 °C [22,23] and *T*_m_ at around 80 °C [24,25], and could potentially be utilized in biomedical applications such as tissue engineering and drug delivery. Thus, the introduction of PL units into PLA could improve the biodegradability of PLA. However, to the best of our knowledge, the synthesis and biodegradation of high molecular weight poly(LA-*co*-PL)s has not been reported systematically, possibly due to the difficulty in their synthesis.

**Scheme 1:**
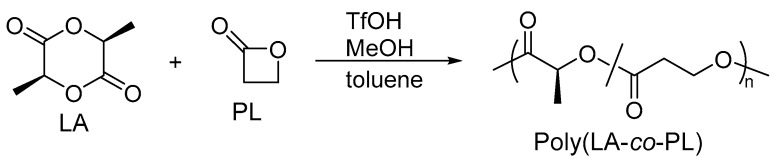
Ring-opening copolymerization of l-lactide (LA) and β-propiolactone (PL) catalyzed by TfOH.

Several catalysts such as distanoxane derivatives [26,27] and salen complexes of aluminum [28,29,30] have been reported to give copolymers of LA with BL. However, those catalysts are not commercially available and more convenient catalysts are desirable. In this work, we studied the copolymerization of LA with PL using trifluoromethanesulfonic acid (TfOH) as a catalyst (Scheme 1) to produce high molecular weight poly(LA-*co*-PL) with different PL-contents, and performed the biodegradation of the obtained copolymers in a compost.

## 2. Results

### 2.1. Copolymerization of LA with PL

**Figure 1:**
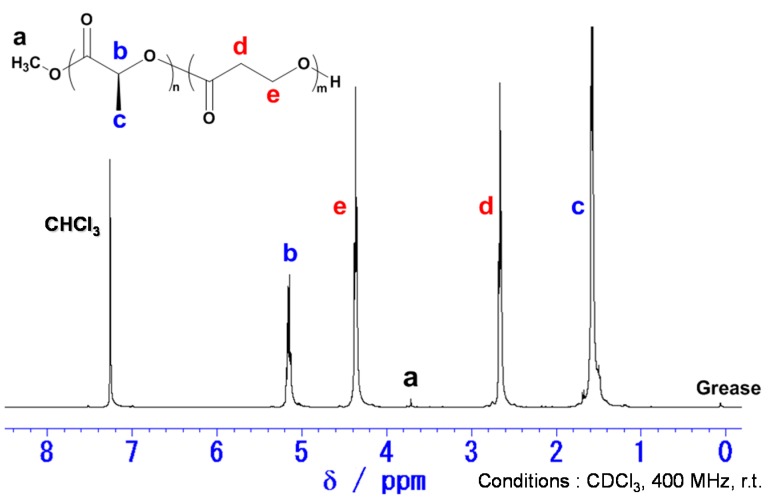
^1^H nuclear magnetic resonance (NMR) spectrum of the poly(LA-*co*-PL) (Table 1, run 1).

Table 1 summarizes the results of the copolymerization of LA and PL at a 1:1 feed molar ratio using TfOH, SmMe(C_5_Me_5_)_2_(THF) (Sm-1), and tin 2-ethylhexanoate (Sn(Oct)_2_) as catalysts. The use of TfOH resulted in the formation of the copolymer with relatively high molecular weight (*M*_n_ = 11,000) in 65% yield, while Sm-1 and Sn(Oct)_2_ did not produced polymers under the present conditions. Figure 1 shows the ^1^H NMR spectrum of the obtained poly(LA-*co*-PL) by TfOH, indicating that the obtained copolymer contained PL and LA units at a 2:1 molar ratio. Thus, TfOH was adopted as a catalyst for the further LA-PL copolymerization experiments.

In order to synthesize high molecular weight copolymers, the feed monomer to initiator ratio was increased to 750/1. Table 2 summarizes the result of LA-PL copolymerization under different feed molar ratios. At feed [LA]_0_/[PL]_0_ of 90/10, the ratio of the initiator (init.) and the catalyst (cat.) was varied in the range of 1/1–1/3 (runs 4–6) for the copolymerization. As a result, the highest conversion was observed at the [init.]/[cat.] of 1/2 (run 5), and thus this condition was applied to further experiments. When the polymerization time was extended from 24 to 48 and 96 h (runs 5,7, and 8), the polymer yield increased with time and produced the copolymer in 98% yield after 96 h. The molecular weights of the resulting polymers also increased with time and reached to *M*_n_ = 55,000 after 96 h, while the molecular weight distribution became rather broad (*M*_w_/*M*_n_ = 1.53). In order to evaluate the effect of PL-content on the polymer properties, the feed [LA]_0_/[PL]_0_ molar ratios were varied from 100:0 to 80:20 (runs 8–12). All runs afforded high molecular weight PLA or poly(LA-*co*-PL) having similar or slightly higher PL-contents in comparison with the feed PL ratio in good yields.

In order to reveal the features of the present LA-PL copolymerization system catalyzed by TfOH, we performed the copolymerization experiments in a short polymerization time (3 h). Then, the reactivity ratios were estimated to be *r*_PL_ = 55.2 and *r*_LA_ = 0.2 by the Fineman–Ross method [31].

### 2.2. Thermal Properties of Poly(LA-co-PL)

**Figure 2:**
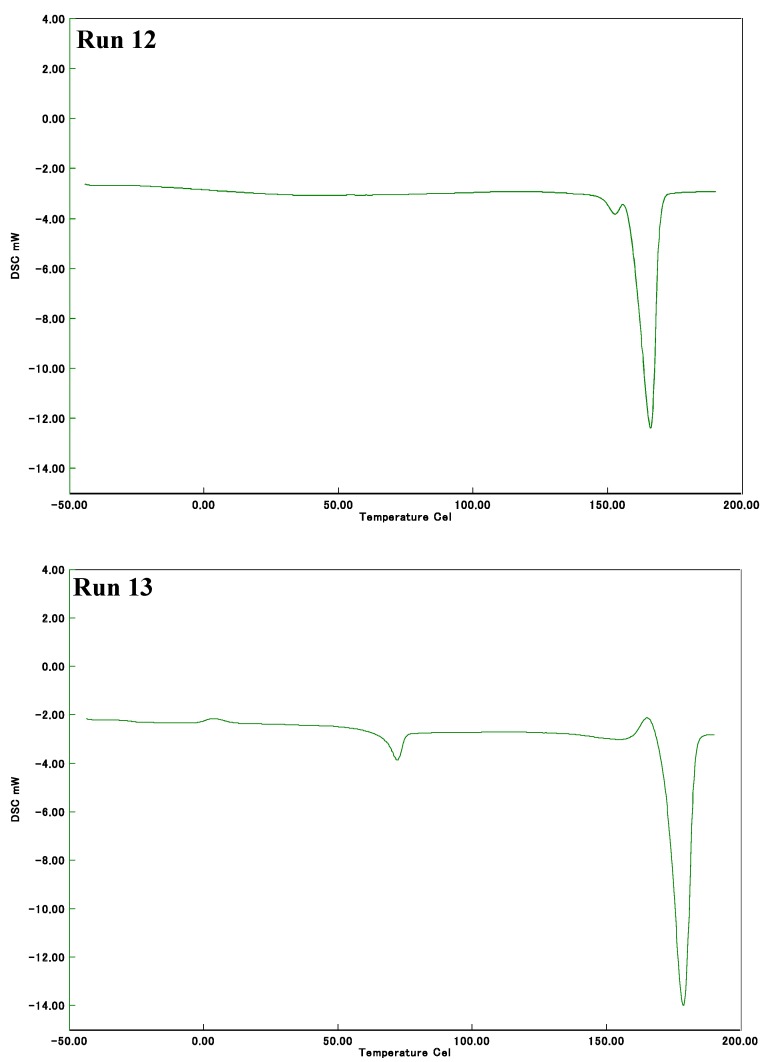
Differential scanning calorimetry (DSC) traces of poly(LA-*co*-PL) (run 12) and a blended sample of poly(l-lactide) and poly(β-propiolactone) homopolymers (run 13).

The thermal properties of the poly(LA-*co*-PL)s were determined by differential scanning calorimetry (DSC) analysis and summarized in Table 3. Upon increasing the PL-content, the *T*_g_ values of the copolymers were rapidly lowered. Each copolymer exhibited only one glass transition. The *T*_m_ and the heat of fusion (Δ*H*_m_) values of the copolymers were gradually lowered with increasing the PL-content. A similar tendency has also been observed for poly(LA-*co*-CL) [12]. The poly(LA-*co*-PL)s (runs 8 and 10–12) showed only one *T*_m_ value corresponding to the PLA segment and no melting transition corresponding to the poly(β-propiolactone) (PPL) segment. This is in sharp contrast to the fact that an 80:20 blended sample of PLA and PPL homopolymers (run 13) showed two melting transitions at 178.3 and 71.6 °C corresponding to *T*_m_ of PLA and PPL homopolymers, respectively (Figure 2).

### 2.3. Biodegradation of Poly(LA-co-PL) in a Compost

**Figure 3:**
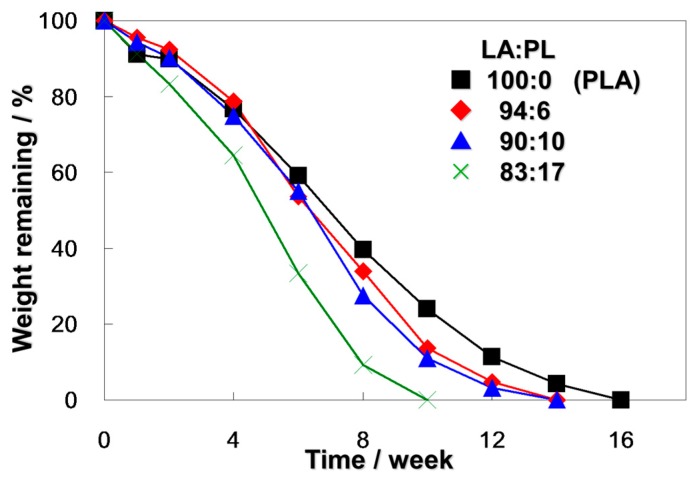
Biodegradation of poly(LA-*co*-PL) and PLA in a compost at 60 °C.

We performed biodegradation tests of the poly(LA-*co*-PL)s as well as PLA in a compost at 60 °C (Figure 3). The PLA homopolymer took 16 weeks for complete weight loss. With increasing PL-contents, the degradation of the copolymers became faster, and the copolymer with a PL-content of 17 mol % was completely degraded within 10 weeks.

## 3. Discussion

**Scheme 2:**
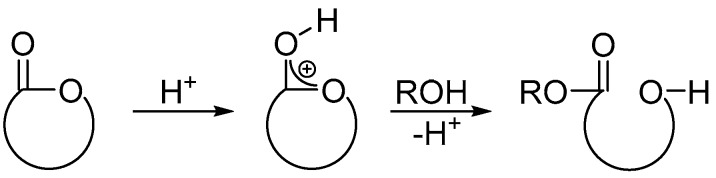
Proposed mechanism of the polymerization of cyclic esters by TfOH.

Among the many catalysts reported for the polymerization of PL, BL, and lactide [16,32,33], we chose TfOH, Sm-1, and Sn(Oct)_2_ for the LA-PL copolymerization in this work. A super acid TfOH was reported to catalyze the polymerization of BL [34]. Rare earth alkoxides such as Y(OMe)(C_5_Me_5_)_2_(THF) [35] and Sm(OEt)(C_5_Me_5_)_2_(THF) [35] were reported to catalyze the polymerization of PL and BL to give polymers with relatively high molecular weights. TfOH [36,37] and rare earth metal complexes similar to Sm-1 [12,38] are also active for the polymerization of lactide. Sn(Oct)_2_ have commonly been used for the polymerization of lactide and middle size lactones such as CL [39,40]. Because TfOH and Sn(Oct)_2_ are used in combination with a protic initiator such as alcohols, we adopted the Sm-1-MeOH system in this study, which should generate Sm(OMe)(C_5_Me_5_)_2_ species [41]. The results shown in Table 1 clearly demonstrated that TfOH was effective for the copolymerization of LA and PL to produce poly(LA-*co*-PL) with relatively high molecular weight (*M*_n_ = 1.1 × 10^4^). Although poly(ethylene glycol)-*block*-poly(d,l-lactide-*co*-PL) has been synthesized by the copolymerization of d,l-lactide and PL catalyzed by Sn(Oct)_2_ in the presence of PEG monomethyl ether (mPEG 550) as an initiator [42], the molecular weight of the copolymer remained very low (*M*_n_ ~ 10^3^). To the best of our knowledge, this is the first example of poly(LA-*co*-PL) with high molecular weight (*M*_n_ > 10^4^). The low activity of the metal catalysts could be attributed to the stable six-membered ring intermediate after the incorporation of PL [35]. In addition, the polymerization of PL by rare earth alkoxide was reported to be accompanied with an elimination side reaction to form an acrylate end-group [43]. The ring-opening polymerization of cyclic esters by TfOH was proposed to proceed in a monomer activation mechanism, as shown in Scheme 2 [37]. We agree with this mechanism and suppose that it is the reason for its lower sensitivity to the ring-size of cyclic ester monomers in the TfOH system in comparison with metal catalyst systems.

At a high feed ratio of monomer to initiator (Table 2), the initiator to catalyst ratio of 1:2 in feed resulted in the highest polymer yield (runs 4–6). In the reported TfOH-catalyzed polymerization of BL, LA, and CL, the initiator to catalyst ratio of 1:1 was applied [34,36,37,44], where the monomer to initiator ratios were rather low. The high monomer to initiator ratio in the present conditions could prefer the initiator to catalyst ratio of 1:2. The molecular weight of the resulting copolymer reached up to *M*_n_ = 5.5 × 10^4^. The increasing molecular weights against polymer yields (runs 5, 7, and 8) suggest that the molecular weights of the resulting polymers can be somewhat controlled by polymerization time and feed monomer to initiator ratio in this polymerization system, although increasing *M*_w_/*M*_n_ with time suggests some side reactions such as trans-esterification.

The PL-contents in the copolymers were higher than those in the feed ratios, indicating the preferential polymerization of PL rather than LA in this copolymerization system. The estimated monomer reactivity ratios indicate that PL is preferentially incorporated into both the PL- and LA-ended propagating chains. The PL-preference in the LA-PL copolymerization should be attributed to the higher ring-strain of PL than that of LA [45]. The large *r*_LA_·*r*_PL_ value of 11 suggests the blocky character of the resulting copolymers.

One glass transition for each copolymer (Table 3) suggests the homogeneous nature of their amorphous phase. Considering the *T*_g_ values of the homopolymers (ca. 60 °C for PLA and ca. −20 °C for PPL [22,23]), the *T*_g_ values of the LA-PL copolymers drastically decreased with increasing PL-contents. This could be attributed to the higher PL-contents of the amorphous phase in the copolymers than those of the whole polymers, because the crystalline phase in the copolymer should be composed of only PLA segments. The *T*_m_ and Δ*H*_m_ values of the copolymers were gently lowered with increasing the PL-content, indicating a mild decrease of crystallinity of the copolymers with increasing PL-contents. These features could come from the blocky nature of the present LA-PL copolymers as mentioned above. On the other hand, the obtained copolymers did not show melting temperatures corresponding to the PPL segment, while the blended sample of PLA and PPL homopolymers (run 13) showed two melting transitions for both the PLA and PPL segments. The *T*_m_ value for the PLA segment and total Δ*H*_m_ values in run 13 is also higher than those of the poly(LA-*co*-PL)s (runs 11 and 12) with similar PL-contents, indicating a decreased crystallinity of the poly(LA-*co*-PL)s. These results imply higher miscibility between the PLA and PPL segments in the poly(LA-*co*-PL)s than that in the blended homopolymers, possibly due to LA-PL conjunctions in the copolymer, and they support that the products from the copolymerization of LA and PL were not merely the mixtures of homopolymers but were in fact copolymers.

Because PLA had been known as a compostable plastic [11,46] and actually used in composting applications, here we adopted biodegradation tests in a compost. In the degradation test (Figure 2), the LA-PL copolymers degraded faster than PLA homopolymer. The degradation rate of the copolymers increased with the PL-contents. Thus, it was demonstrated that the incorporation of PL units into PLA enhanced its biodegradability. The improved degradability of the copolymers could come from the inherent degradability of the PPL segment and/or the decreased crystallinity of the copolymers.

## 4. Materials and Methods

### 4.1. General

All the polymerization reactions were performed under a dry nitrogen atmosphere using standard Schlenk techniques. ^1^H NMR spectra were recorded on a JNM-LA400 spectrometer (400 MHz for ^1^H nuclei) (JEOL, Tokyo, Japan). Chemical shifts of ^1^H NMR spectra in chloroform-*d* were calibrated by using the signals for residual chloroform (δ = 7.26 ppm). Molecular weights and polydispersities of the polymers were determined by gel permeation chromatography (GPC) measurements on a Tosoh GPC system (SC-8010) (Tosoh, Tokyo, Japan) equipped with an refractive index detector. GPC curves were calibrated using standard polystyrenes. THF was used as an eluent at a flow rate of 1.0 mL/min at 40 °C. The melting temperature (*T*_m_), heat of fusion (Δ*H*_m_), and glass transition temperature (*T*_g_) of the polymers were measured on a differential scanning calorimetry (DSC) using a DSC 6220 apparatus (Seiko, Tokyo, Japan). The heating rate was 10 °C/min in a nitrogen stream. The thermodegradation behavior was measured by thermogravimetric analysis on a TG/DTA 6300 apparatus (Seiko, Tokyo, Japan).

### 4.2. Materials

Dehydrated tetrahydrofuran (Kanto Chemical, Tokyo, Japan) was further purified by distillation from Na-benzophenone under nitrogen prior to use. Toluene was purified by distillation from sodium-benzophenone. Each solvent was stored over sodium. Chloroform was dried over CaH_2_ overnight and then distilled. Methanol (Kanto Chemical) and PhCH_2_OH (Wako Pure Chemical, Tokyo, Japan) was distilled, and stored over activated molecular sieves (3A). LA and PL were purchased from Tokyo Chemical Industry (Tokyo, Japan). LA was sublimated under nitrogen before use. PL and TfOH (Sigma-Aldrich Japan, Tokyo, Japan) were distilled under reduced pressure before use. Sn(Oct)_2_ was purchased from Sigma-Aldrich and used without further purification. SmMe(C_5_Me_5_)_2_(THF) (Sm-1) was synthesized according to Reference [47].

### 4.3. Copolymerization of LA and PL Catalyzed by TfOH

Certain amounts of TfOH and methanol were added to a mixture of prescribed amounts of l-lactide and β-propiolactone in toluene. The mixture was stirred at 50 °C for a given time. The mixture was poured into an excess amount of methanol to precipitate the polymer, which was collected by centrifugation and dried in vacuo.

### 4.4. Copolymerization of LA and PL Catalyzed by Sm-1

Sm-1 (0.02 mmol) was reacted in situ with one equivalent of methanol in toluene (1 mL). A solution of LA (0.72 g, 5.0 mmol) and PL (0.31 mL, 5.0 mmol) in toluene (4 mL) was added to the reaction mixture of Sm-1 and methanol in toluene, and the mixture was stirred at 0 °C for 12 h. The mixture was poured into excess methanol to precipitate the polymer, however, no precipitation appeared.

### 4.5. Copolymerization of LA and PL Catalyzed by Sn(Oct)_2_

A solution of LA (0.0.73 g, 5.0 mmol) and PL (0.31 mL, 5.0 mmol) in toluene (5 mL) was added to Sn(Oct)_2_ (3.2 μL, 0.01 mmol) and PhCH_2_OH (4.1 μL, 0.04 mmol) in toluene. The mixture was stirred at 100 °C for 24 h. Then, the resulting mixture was poured into excess methanol to precipitate the polymer, however, no precipitation appeared.

### 4.6. Degradation of the Polymers by a Compost

The degradation tests were carried out according to the literatures [38,48]. Commercially available effective microorganism (EM)-fermented solution (30 mL) containing *Rhodospirillum*, *Rhodopseudomonas*, *Pseudomonas*, *Micrococcus*, *Bacillus*, *Lactobacillus*, *Streptococus*, *Saccharomyces*, *Aspergillus*, *Penicillium* etc. and theriaca syrup (40 mL) was added to 2000 mL of water, and this solution was sprayed on the mixture of rice hulls (5 kg) and rice bran (15 kg). The resulting material was wrapped with a polyethylene film and then dried in the shade for 1 day. The content of water was evaluated by the weight loss of the samples after heating them to 200 °C. The poly(LA-*co*-PL)s and PLA were shaped into films by solution casting from CHCl_3_. The film samples were sealed in polyethylene mesh and held in the resulting compost for a fixed time. The evaluation of the biodegradation was carried out by measuring the weight loss with the compost.

## 5. Conclusions

In conclusion, LA-PL copolymerization was studied using several catalysts. TfOH was found to be an effective catalyst for the LA-PL copolymerization to afford poly(LA-co-PL) with high molecular weight (*M*_n_ ~ 50,000) and various PL-contents (6–67 mol%). The obtained copolymers exhibited rapidly decreasing *T*_g_ values with increasing PL-contents, while their *T*_m_ and Δ*H*_m_ values gradually decreased with increasing PL-contents. Biodegradation test of the copolymers in compost demonstrated the biodegradability of the copolymers increasing with PL-contents.

## Figures and Tables

**Table 1:** Comparison of catalysts for the copolymerization of LA and PL ^1^.

Run	Catalyst	[LA]_0_/[PL]_0_/[init.]/[cat.]	Temp. (°C)	LA-Conv. ^2^ (%)	PL-Conv. ^3^ (%)	*M*_n_^4^ (10^3^)	*M*_w_/*M*_n_^4^	PL-Cont. ^5^ (mol%)
1 ^6^	TfOH	125/125/1/1	50	48	98	11	1.2	67
2 ^6,7^	Sm-1	250/250/1/1	0	0	0	-	-	-
3 ^8^	Sn(Oct)_2_	500/500/4/1	100	0	0	-	-	-

^1^ Conditions: Solvent = toluene (5 mL), LA_0_ = PL_0_ = 5 mmol, time = 24 h; ^2^ Conversion of LA calculated from polymer yield and the composition of the resulting copolymer; ^3^ Conversion of PL calculated from polymer yield and the composition of the resulting copolymer; ^4^ Determined by gel permeation chlomatography (GPC) in tetrahydrofuran (THF) calibrated with standard polystyrenes; ^5^ PL-content determined by ^1^H NMR analysis; ^6^ Init. = CH_3_OH; ^7^ Time = 12 h; ^8^ Init. = PhCH_2_OH.

**Table 2:** Copolymerization of LA and PL by TfOH ^1^.

Run	[LA]_0_/[PL]_0_	[init.]/[cat.]	Time (h)	LA-Conv. ^2^ (%)	PL-Conv. ^3^ (%)	*M*_n_^4^ (10^3^)	*M*_w_/*M*_n_^4^	PL-Cont. ^5^ (mol %)
4	90/10	1/1	24	32	>99	10	1.11	27
5	90/10	1/2	24	67	>99	26	1.16	18
6	90/10	1/3	24	64	>99	30	1.17	15
7	90/10	1/2	48	86	96	37	1.11	11
8	90/10	1/2	96	98	98	55	1.53	10
9	100/0	1/2	96	95	-	41	1.59	0
10	95/5	1/2	96	89	>99	35	1.73	6
11	85/15	1/2	96	80	93	32	1.51	17
12	80/20	1/2	96	68	>99	25	1.25	27

^1^ Conditions: Solvent = toluene (5 mL), LA_0_ + PL_0_ = 10 mmol, (LA_0_ + PL_0_)/init. = 750/1, init. = MeOH, temp. = 50 °C; ^2^ Conversion of LA calculated from polymer yield and the composition of the resulting copolymer; ^3^ Conversion of PL calculated from polymer yield and the composition of the resulting copolymer; ^4^ Determined by GPC in THF calibrated with standard polystyrenes; ^5^ Determined by ^1^H NMR analysis.

**Table 3:** Thermal properties of poly(LA-*co*-PL) ^1^.

Run	PL-Content	*T*_g_ (°C)	*T*_m_ (°C)	Δ*H*_m_ (J/g)
9	0	62	172	55
10	6	45	170	53
8	10	41	167	49
11	17	24	165	45
12	27	11	166	42
13 ^2^	20	-	72,178	4,46

^1^ Determined by DSC analysis; ^2^ A mixture of PLA and PPL homopolymers.
